# The Correlation Between Gross Domestic Product Per Capita and the Success Rate of Assisted Reproductive Technologies Worldwide

**DOI:** 10.5935/1518-0557.20210078

**Published:** 2022

**Authors:** Jie Dong, Song Yan, Chenxi Qian, Xiaohong Wang

**Affiliations:** 1 Reproductive Medical Center, Department of Obstetrics and Gynecology, Tangdu Hospital, Air Force Medical University, Xi'an, Shaanxi Province, China

**Keywords:** GDP, assisted reproductive technologies, pregnancy rate, delivery rate

## Abstract

**Objective:**

Although there has been increased utilization of assisted reproductive technologies (ART) in the world, there is no conclusive definition about the relationship between the success rate of ART and national wealth.

**Methods:**

In this study, using the data from the International Committee for Monitoring Assisted Reproductive Technologies (ICMART), we sought to determine whether there is a correlation between the success rate of ART (represented by pregnancy and delivery rates) and national wealth represented by the gross domestic product (GDP) per capita. Moreover, to further understand the effect of GDP per capita on ART effectiveness, we analyzed the association between ART success rate and GDP per capita in 50 US states.

**Results:**

Our data showed that the number of ART treatment cycles increased as the GDP per capita increased. However, we found a negative correlation between ART success rates and GDP per capita in ICMART countries, although no correlation was seen in the US states. Using rough estimation, we derived that the success rate of ART was not related to GDP per capita in the ICMART countries with a GDP per capita greater than USD 13,000.

**Conclusions:**

In conclusion, for the first time, we showed that when the GDP per capita of an economic territory reaches (or exceeds) USD 13,000, ART pregnancy and delivery rates were not associated with GDP per capita, and ART success rates remained stable.

## INTRODUCTION

Since the world's first "test-tube" baby was born in 1978, assisted reproductive technologies (ART) have developed rapidly and been widely used all over the world. Commonly used ART includes in vitro fertilization (IVF), intracytoplasmic sperm injection (ICSI), and frozen embryo transfer (FET). It has been reported that over eight million babies have been born via ART (European Society of Human Reproduction and Embryology, 2018). Today, in some countries, ART-conceived infants account for between 1.8% to 5% of all infants (Sunderam *et al*., 2019; European IVF-monitoring Consortium (EIM)‡ for the European Society of Human Reproduction and Embryology, 2020). However, despite the advances in ART, its success rate per treatment cycle remains low. Based on the ART data of 69 countries, the ICMART has reported that the delivery rate via IVF/ICSI (combined) and FET is 19.8% and 22.1%, respectively (de Mouzon *et al*., 2020). In the United States, official data shows that the live birth rate per cycle via IVF/ICSI (combined) and FET for non-donor women aged less than 35 years is 31.0% and 49.4%, respectively; and the rate gets lower for older women (Sunderam *et al*., 2019).

Multiple factors have been associated with the low percentages of live births, including parent age, infertility-related factors, and ART manipulation itself (Tarín *et al*., 2014). The quality and policies of a healthcare system may also influence ART success rates. For example, a superior healthcare system that is funded properly could mean better ART services. Commonly, developed countries/regions provide superior healthcare systems and funds to support ART treatments to infertile couples. Hence, ART treatments in these countries/regions are relatively affordable, especially to sub-fertile couples, and may achieve a high success rate. Surprisingly, a study performed by Lass *et al*. (2019) refutes this postulation. Although the study found a robust positive correlation between the number of IVF cycles performed and GDP per capita, it also showed that both pregnancy and delivery rates via IVF were inversely correlated with the GDP per capita. The researchers assumed that the unexpected findings were associated with inadequate comprehensive data collection (such as age distribution, causes of infertility, and quality control level of embryo handling - due to objective limitations). Based on their limited data, however, it appears that developed countries do not have a high IVF success rate.

Despite the gradual increase in the usage of other regular ART treatments (such as ICSI and FET) (European IVF-monitoring Consortium (EIM)‡ for the European Society of Human Reproduction and Embryology, 2020), there has been no conclusive answer about the relationship between the success rate of ICSI/FET cycles and national wealth. Therefore, using data from the ICMART, we investigated whether there is a correlation between pregnancy/delivery rates from ICSI/FET cycles and national wealth represented by the GDP per capita. Moreover, to increase the understanding of the effect of national wealth on the effectiveness of ART, we analyzed the relationship between ART success rate and GDP per capita in 50 states of the US.

## MATERIALS AND METHODS

All original data analyzed were collected from published papers and materials. We used the latest ICMART ART data (including treatment cycles, pregnancy, and delivery rates) (de Mouzon *et al*., 2020) and the GDP per capita data of all countries in the world in 2012. Given the potential influence of confounding factors, such as demographic and cultural characteristics, we increased our scope to analyze ART data from 50 US states. To maintain consistency with the 2012 ICMART data, we collected the 2012 ART data of individual states released by the US Health Department in 2015 (Sunderam *et al*., 2015), and the 2012 GDP per capita of each state (Brooks *et al*., 2013). The original data are populated in the Supplementary Tables 1 and 2.

### Statistical Analyses

Data analyses were performed using IBM SPSS Statistics 23.0 (IBM software, 81 NY, USA). Graphics were drawn using Prism 8 (GraphPad Software, CA, USA). Normal variables are shown as means and standard deviation, and non-normal variables are shown as medians with percentile values. We used Spearman's rank correlation coefficient to investigate the relationship between GDP per capita (unit: 10 thousand dollars) and ART cycles, and employed Pearson's correlation coefficient (CC) to investigate the relationship between GDP per capita and ART success rate after logarithmic processing of GDP per capita (shown as LogGDP). A *p*-value < 0.05 (two-tailed) was considered significant.

## RESULTS

1. The number of ART treatment cycles increased as the GDP per capita increased based on 2012 ICMART data.

**Figure 1:**
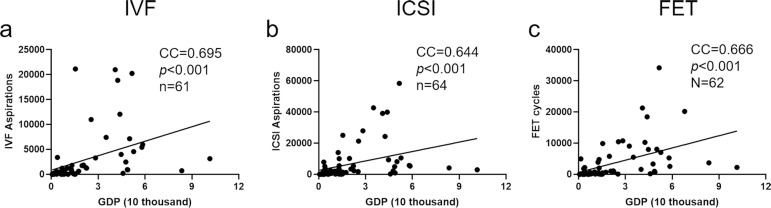
The correlation between GDP per capita (USD 10,000) and number of ART treatment cycles from 2012 ICMART data. a: correlation between GDP per capita and IVF cycles; b: correlation between GDP per capita and ICSI cycles; c: correlation between GDP per capita and FET cycles. CC: correlation coefficient.

Considering the different ARTs corresponding to different applicable conditions, we analyzed the correlation between the GDP per capita and the number of cycles of IVF, ICSI, and FET, respectively. Based on ICMART data, we calculated the median of GDP per capita to be 1.53 (0.74-4.36), and the median of the number of cycles of IVF, ICSI, and FET cycles to be 920.0 (277.3-3356.5), 2529.0 (631.5-8988.3), and 713.5 (118.8-5117.0), respectively. As shown in Figure 1, there was a significant positive correlation between GDP per capita and the number of ART cycles in its various forms, including IVF (Figure 1a, CC=0.695, *p*<0.001), ICSI (Figure 1b, CC=0.644, *p*<0.001), and FET (Figure 1c, CC=0.666, *p*<0.001). The results suggest that ART treatment cycles increase with an increase in the GDP per capita of a country. 

2. The pregnancy and delivery rates both in IVF and ICSI aspiration cycles were negatively correlated with GDP per capita based on 2012 ICMART data. 

**Figure 2:**
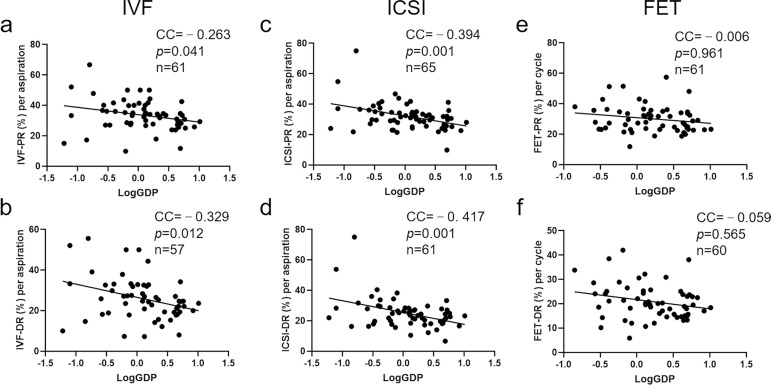
The correlation between LogGDP and pregnancy/delivery rates in ART cycles from 2012 ICMART data, respectively. a: pregnancy rate in IVF cycles (IVF-PR). b: delivery rate in IVF cycles (IVF-DR); c: pregnancy rate in ICSI cycles (ICSI-PR). d: delivery rate in ICSI cycles (ICSI-DR); e: pregnancy rate in FET cycles (FET-PR). f: delivery rate in FET cycles (FET-DR). CC: correlation coefficient.

Next, we tested the correlation between pregnancy and delivery rates in the three regular ART cycles (IVF, ICSI, and FET) and LogGDP using Pearson's correlation coefficient. The results showed that in IVF cycles, both pregnancy (Figure 2a, CC=-0.263, *p*=0.041) and delivery rates (Figure 2b, CC=-0.329, *p*=0.012) were inversely correlated with LogGDP of ICMART countries. Similarly, for ICSI cycles, there was a significant negative correlation between pregnancy (Figure 2c, CC=-0.394, *p*=0.001) and delivery rates (Figure 2d, CC=-0.417, *p*=0.001) and logGDP. However, in FET cycles, we found no significant correlation between pregnancy (Figure 2e, CC=-0.006, *p*=0.961) and delivery rates (Figure 2f, CC=-0.059, *p*=0.565) and LogGDP. The data herein indicate that the success rate in IVF and ICSI cycles declines with an increase in the GDP per capita of a country.

3. There was no correlation between ART success rate and GDP per capita based on the 2012 ART data of the 50 US states. 

**Figure 3:**
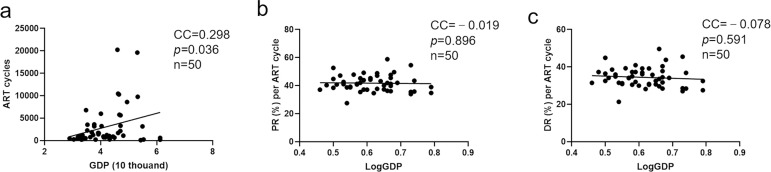
The correlation between GDP per capita and number of ART treatment cycles from 2012 USA data (a). The correlation between LogGDP and pregnancy rate (b) and delivery rate (c) in ART cycles, respectively. CC: correlation coefficient.

Considering the potential influence of complex demographic, geographical, and cultural backgrounds and their effects on the correlation between GDP per capita and ART success rate, we used data from 50 US states with relatively fewer confounding factors to analyze the relationship between GDP per capita and ART success rate. First, we found that the average pregnancy and delivery rates were 41.6±5.9% and 34.2±5.4% in ART cycles, respectively. Subsequently, we analyzed the correlation between ART cycles (excluding FET cycles) and GDP per capita, and the results showed that ART procedures increased with an increase in GDP per capita (Figure 3a, CC=0.298, *p*=0.036). However, we found that there was no significant relationship between pregnancy (Figure 3b, CC=-0.019, *p*=0.896) and delivery rates (Figure 3c, CC=-0.078, *p*=0.591) and LogGDP. 

4. There may be a boundary point to link the correlation between ART success rate and GDP per capita. 

Based on the analysis of 2012 ICMART data and 2012 ART data of 50 US states, we found that there was a negative link between ART success rate and GDP per capita in ICMART countries, although no such correlation was found in the US states. Because the median GDP per capita of the US states [5.38 (4.54-6.15)] in 2012 was much higher than that of ICMART countries [1.53 (0.74-136 4.36)], we speculated that ART success rate might have nothing to do with GDP per capita after the GDP per capita of an economic territory reaches a certain level. In other words, there might be a correlation between ART success rate and GDP per capita within a certain GDP per capita range in ICMART members. To estimate the boundary point, we compared the correlative variables at different GDP per capita levels in determining the association between the ART success rate and GDP per capita. As shown in Table 1, when the GDP per capita was set at ≥ USD 12,000, we found a significant negative correlation between pregnancy rate only in IVF cycles and GDP per capita (CC=-0.380, *p*=0.026); however, when the GDP per capita was above USD 13,000, there was no statistically significant correlation between pregnancy/delivery rates from IVF and ICSI cycles and GDP per capita. The results indicated that there might be no correlation between ART success rate and GDP per capita when the GDP per capita of an economic territory is greater than USD 13,000.

**Table 1.** Spearman correlation analysis between GDP per capita and ART pregnant and delivery rates when GDP per capita is ≥1.2 or ≥1.3, respectively.

GDP per capita ≥1.2 (LogGDP ≥0.08)			GDP per capita ≥1.3 (LogGDP ≥0.11)			
	CC	*p* value		CC	Mean (deviation)	*p* value
IVF-PR n=34	-0.380	0.026	IVF-PR n=31	-0.320	31.7±6.85	0.079
IVF-DR n=32	-0.225	0.216	IVF-DR n=30	-0.133	23.0±7.4	0.483
ICSI-PR n=35	-0.291	0.089	ICSI-PR n=32	-0.205	29.3±4.6	0.260
ICSI-DR n=33	-0.090	0.620	ICSI-DR n=31	0.039	21.5±4.9	0.834

## DISCUSSION

In this study, we investigated the correlation between the ART success rate of a country (represented by pregnancy and delivery rates) and its wealth (represented by the GDP per capita). Relying on data from 2012 ICMART members and 50 US states, we found different results: there was a negative correlation between ART success rate and GDP per capita in ICMART countries, but no such correlation existed in the 50 US states. Using rough estimation, we found that ART success rate was not related to GDP per capita in ICMART countries when the GDP per capita was greater than USD 13,000. Therefore, we conclude that when the GDP per capita of a country or economic territory exceeds USD 13,000, their ART pregnancy and delivery rates might remain at a stable level.

An estimated more than 48 million people are affected by infertility worldwide (Mascarenhas *et al*., 2012). Many IVF clinics are opening across the world to help infertile couples have babies. Data from the IVF-Worldwide website (www.ivf-worldwide.com) show that more than 3,000 IVF clinics are operational, but ART treatments are still inadequate in many parts of the world, particularly in developing countries (Inhorn & Patrizio, 2015). 

However, the prevalence of infertility worldwide is comparable across low-, middle-, and high-income countries (Asemota & Klatsky, 2015). Accordingly, wealthier countries show more utilization of ART treatments. According to our data, there is a higher ART utilization rate (including IVF, ICSI, and FET cycles) in countries/regions with a higher GDP per capita. In their study, Lass *et al*. (2019) also showed a positive correlation between the utilization rate of IVF and the GDP per capita of countries/regions. This can be attributed to the affordability of receiving ART treatments in these countries/regions. As such, governments across the world should begin and/or continue/increase support for ART treatments, as occurs in Nordic countries (Lass *et al.,* 2019). 

Generally, people expect developed countries/regions to have high success rates of ART treatments. However, data from ICMART member countries showed a strong negative correlation between GDP per capita and IVF/ICSI treatment success rates in developed countries/regions. Noticeably, countries with a lower GDP per capita had higher pregnancy and delivery rates in IVF/ICSI treatments. It is difficult to determine the cause of this startling relationship owing the limitations of data availability. A few potential factors may explain this relationship. For example, it is well known that female age is remarkably associated with fertility and ART success rate; women aged over 35 years have reduced fecundity and, therefore, lower pregnancy rates especially via ART treatments (Howles *et al*., 2006). Evidence shows that, in developed countries, women prefer to have children late in their lives (Mills *et al*., 2011). To allow time to finish their education, pursue careers, or attain financial independence, many women in developed countries delay starting families until they are 40 years or older (Mills *et al*., 2011; Armstrong & Akande, 2013). This phenomenon means that reproductive health clinics are treating a higher number of older infertile women with more complicated gynecological conditions, such as diminished ovarian reserve, endometriosis, and adenomyosis, thereby, the reduced ART success rate in these countries. In Japan, for example, a country with a serious aging problem (Nishi *et al*., 2020), the pregnancy and delivery rates are much lower even by IVF (11.7% and 8%, respectively) and ICSI (9.8% and 6.6%, respectively) (Supplementary Table 1). Another factor affecting the ART success rate may be the number of treatment cycles. As our data show, the ART treatment cycles grow as the GDP per capita of an economic territory increases. Statistically, the success rate of ART treatments calculated from a larger number of cases should be closer to the overall rate. Thus, the pregnancy and delivery rates in IVF/ICSI cycles in low-income countries (such as Ghana, Mali, and Nicaragua) might not adequately reflect their ART success rate owing to the relatively small number of IVF/ICSI cycles.

Additionally, the different cultural backgrounds across regions may affect the correlation between GDP per capita and ART success rate. However, it is difficult to test and verify their role in ART success rate owing to data unavailability. Therefore, to reduce the influence of cultural backgrounds, we used data from 50 US states with relatively lower confounding factors to analyze the relationship between GDP per capita and ART success rates. Surprisingly, we found no significant correlation between GDP per capita and ART pregnancy/delivery rates based on the US CDC data. Given the high GDP per capita of the US states, we hypothesized that ART success rates might have nothing to do with GDP per capita when the GDP per capita of an economic territory reaches a certain level. Subsequently, by analyzing data of ICMART members, we estimated this GDP per capita threshold to be USD 13,000; in other words, ART pregnancy and delivery rates were more stable (20%-30%, Table 1) in the regions where the GDP per capita was greater than USD 13,000. Notably, however, the success rate of ART is still not satisfactory worldwide. The latest data from the European Society of Human Reproduction and Embryology (ESHRE) show that the live birth rate per aspiration is ~20% in both IVF and ICSI cycles (European IVF-monitoring Consortium (EIM)‡ for the European Society of Human Reproduction and Embryology, 2020). It appears that the era of improving ART success rate is moving into a bottleneck period as ART has been developing for 40 years. 

This study presents limitations about the quality of data due to the lack of basic characteristics, such as the age distribution of patients, infertility factors, and the quality control standards of reproductive health clinics. It was difficult to collect such detailed information owing to inaccessibility to the reporting registry system. Moreover, only about a third of the countries in the world publish their ART data to ICMART. Currently, a large number of reproductive health clinics are opening in Asian countries, such as China, Thailand, and Vietnam. It will be crucial to obtain ART registry data from these countries. Additionally, owing to the limited ICMART data from a rather small number of countries, we were not able to obtain a fitting curve to show the correlation between GDP per capita and ART success rate (data not shown). We provided only a rough estimate (~USD 13,000) of the boundary point. Therefore, there is a possible bias in obtaining this critical value. 

In summary, our study, for the first time, revealed that the success rate of ART might attain a stable level when the national or regional wealth reaches a certain level (GDP per capita of USD 13,000). Despite the low number of ART treatment cycles in developing countries, the ART success rate of these countries is not inferior to that of developed countries. Nevertheless, further studies analyzing more ART data collected from more countries across the world are necessary to understand the influence of socioeconomic status on the overall effectiveness rate of ART treatment. 

## ABBREVIATIONS

ART: Assisted Reproductive Technologies

CC: Correlation Coefficient

FET: Frozen Embryo Transfer

GDP: Gross Domestic Product

ICSI: Intracytoplasmic Sperm Injection

ICMART: International Committee for Monitoring Assisted Reproductive Technologies

IVF: In Vitro Fertilization (IVF)

## Appendix

**Supplementary Table 1.** GDP per capita of countries and the reported data about ART cycles and pregnant/delivery rates in 2012.

Country	GDP PER CAPITA (10 thousand dollars)	IVF			ICSI			FET		
		IVF aspirations	IVF-PR (%)	IVF-DR (%)	ICSI aspirations	ICSI-PR (%)	ICSI-DR (%)	FET cycles	FET-PR (%)	FET-DR (%)
Albania	0.4247	0	NA	NA	204	41.7	33.8	39	51.3	38.5
Argentina	1.31	504	35	27	5515	25.1	18.4	1971	27.8	20.2
Australia	6.8	NA	NA	NA	NA	NA	NA	20181	29.1	22.5
Austria	4.86	920	34.7	NA	4919	31.3	NA	955	34.6	13.3
Belarus	4.16	1229	39.8	27.3	659	46.7	34.4	107	26.2	13.1
Belgium	4.47	3996	28.7	21.1	9277	25.5	18.4	7996	26.4	18.9
Benin	0.08	9	33.3	33.3	92	37	28.3	0	NA	NA
Bolivia	0.26	148	36.7	25.9	62	36.1	27.9	14	35.7	28.6
Brazil	1.24	1070	32.4	28.4	13937	31	25.2	3895	32.3	25.3
Bulgaria	0.74	593	27.3	17.9	587	21.4	15.6	573	33.5	26.5
Cameroon	0.14	204	17.2	14.7	202	21.8	16.3	NA	NA	NA
Canada	5.27	4552	33.4	24.5	10494	35.8	27.1	7028	32.4	23.7
Chile	1.54	131	44.3	32.8	1321	35	27.2	470	35.7	25.7
Colombia	0.81	293	35	27.5	622	34.2	26.5	165	23	18.2
Croatia	1.32	1811	26.7	7.2	2736	24.2	10.5	131	23.7	10.7
Czech Republic	1.97	1739	17.8	14.2	10147	33.4	24.4	5789	31.7	20.1
Denmark	5.85	5970	25	22.1	5278	25.2	22.7	2566	22.6	19.5
Dominican Republic	0.68	42	9.8	7.3	35	22.9	20	3	NA	NA
Ecuador	0.57	216	43.7	37.9	324	29	21.8	121	34.7	28.9
Egypt	0.32	0	NA	NA	7848	35	17.9	1819	23.1	10.2
Estonia	2.32	606	27.7	22.8	1188	28.7	23.5	634	18.8	12
Finland	4.77	2475	28.6	22.1	2143	25.7	20.8	3319	24.3	18
France	4.09	20995	23.8	19.2	39079	24	19.6	21296	18.7	14.7
Germany	4.39	12047	27.2	17.8	39911	26.6	18.1	18466	21	13.2
Ghana	0.16	18	66.7	55.6	9	75	75	0	NA	NA
Greece	2.22	1329	32.8	15.7	5343	32.2	17.2	1079	35	17.1
Guatemala	0.34	38	27	18.9	62	29.5	19.7	7	42.9	14.3
Hungary	1.29	920	34.5	NA	3502	31.7	NA	398	31.7	NA
Iceland	4.59	199	25.6	18.1	206	22.8	19.9	186	19.9	15.1
India	0.14	NA	NA	NA	NA	NA	NA	4959	38	33.8
Indonesia	0.37	519	NA	NA	2635	40.4	40.4	427	24.1	24.1
Ireland	4.89	904	34.4	24.7	922	32	24.4	670	24.9	15.1
Israel	3.25	NA	NA	NA	NA	NA	NA	9044	24.7	15.7
Italy	3.51	7397	23.9	15.3	42690	21.8	14	5496	22.4	14.4
Japan	4.86	77370	11.7	8	122962	9.8	6.6	116023	33.7	23
Kazakhstan	2.08	1188	37.5	24.7	1059	41.8	28.3	436	36.5	24.3
Lebanon	0.8	3	33.3	33.3	945	43.9	38.3	17	11.8	5.9
Lithuania	1.43	103	41.7	32	46	32.6	21.7	24	33.3	20.8
Mali	0.08	53	52.1	52.1	101	54.8	53.8	23	NA	NA
Mexico	1.02	1222	41.4	32.9	2017	32.4	26.4	757	27.3	22.1
Moldova	0.3	429	36.1	32.6	667	38.8	36	43	23.3	18.6
Montenegro	0.66	2	50	50	504	29.4	24.8	14	21.4	21.4
Morocco	0.29	126	27	NA	433	29.8	NA	NA	NA	NA
Netherlands	5.01	7139	28.2	19.4	8122	29.8	21.8	8063	23.2	15.7
New Zealand	4	NA	NA	NA	NA	NA	NA	1572	34	25.1
Nicaragua	0.18	46	47.8	39.1	41	36.6	31.7	NA	NA	NA
Nigeria	0.27	272	40.1	18.2	1121	27.1	16.4	54	27.8	24.1
Norway	10.15	3131	29.3	23.6	2925	28	23.2	2208	23.2	18.4
Panama	1.07	7	50	50	192	40.1	28.5	72	43.1	30.6
Peru	0.65	298	28.6	23.5	875	22.9	17.7	231	51.5	42
Poland	1.31	450	30	20.9	10017	34.7	26.4	4736	27.8	19.6
Portugal	2.06	1838	34.3	26.5	3385	28.4	21.8	1011	23.1	16
Romania	0.85	627	40	31.9	908	31.6	25.3	333	21	12
Russia	1.54	21144	34.1	24.3	25062	30.1	21.2	9880	32.1	18.7
Saudi Arabia	2.52	0	NA	NA	1670	33	29.7	68	57.4	30.9
Serbia	0.6	510	35.3	27.1	1386	34.6	28.6	NA	NA	NA
Slovenia	2.26	1231	31.8	25.3	2278	25.5	20.5	801	25.5	20.3
South Africa	0.75	994	28.3	NA	2423	27.9	NA	541	29.6	NA
South Korea	2.55	10980	34.6	12.2	21280	29.6	12.2	10441	36.1	14.6
Spain	2.83	3277	33.5	19.4	27926	31	18.1	10744	31.7	17.9
Sweden	5.8	5437	30.8	24.7	5695	30.6	24.9	5244	28.7	22.9
Switzerland	8.35	710	25.8	20	4126	22.6	16.6	3697	22.9	17
Togo	0.06	20	15	10	53	24	22	5	0	0
Tunisia	0.42	378	36	33.1	3581	33.6	29.4	757	27.3	21.1
UK	4.25	18853	31	27	24299	31.8	27.8	10253	27.4	23.8
Ukraine	0.39	3383	41.6	29.9	5116	37.4	29.9	2173	36.3	25.1
Uruguay	1.52	20	50	44.4	233	31.6	26.8	56	30.4	19.6
USA	5.16	20233	42.5	34.2	58397	41.2	33.3	34197	48.1	38.1
Venezuela	1.3	369	40	32.6	184	33.7	23.7	118	41.5	32.2

**Supplementary Table 2.** GDP per capita of USA states and the reported data about ART cycles and pregnant/delivery rate in 2012.

States	GDP PER CAPITA (10 thousand dollars)	ART cycles	PR (%) Pregnancies/ART	DR (%) Pregnancies/ART
Alabama	3.26	873	40.8	34.1
Alaska	6.12	207	34.8	27.5
Arizona	3.52	2261	39.2	30.9
Arkansas	3.18	492	38.4	32.5
California	4.6	20241	41.9	33.6
Colorado	4.62	1705	58.8	49.6
Connecticut	5.49	3148	35.7	28.4
Delaware	6.12	585	39.0	32.5
District of Columbia	14.6	1228	32.4	25.1
Florida	3.48	6785	38.7	31.5
Georgia	3.77	3176	45.2	37.2
Hawaii	4.44	886	40.1	32.5
Idaho	3.19	399	52.6	44.9
Illinois	4.62	10449	36.3	29.6
Indiana	3.91	1729	37.2	32.0
Iowa	4.22	1225	48.8	39.2
Kansas	4.11	689	45.3	39.2
Kentucky	3.35	1172	41.0	34.9
Louisiana	4.31	1013	47.1	30.9
Maine	3.46	506	27.5	21.3
Maryland	4.67	5770	39.8	31.7
Massachusetts	5.32	9754	35.5	28.6
Michigan	3.53	3532	38.8	31.3
Minnesota	4.7	2130	47.7	40.4
Mississippi	2.89	461	37.1	31.5
Missouri	3.68	1847	41.2	34.1
Montana	3.32	210	47.1	38.6
Nebraska	4.49	649	37.3	30.7
Nevada	4.1	914	45.2	37.0
New Hampshire	4.3	817	37.7	30.5
New Jersey	4.94	8590	41.9	34.1
New Mexico	3.39	359	42.6	35.9
New York	5.31	19618	34.0	27.0
North Carolina	4.03	3219	44.2	36.1
North Dakota	5.53	239	43.5	36.8
Ohio	3.77	3601	42.0	35.0
Oklahoma	3.63	759	47.8	39.3
Oregon	4.81	1104	49.6	43.8
Pennsylvania	4.01	5984	37.1	30.0
Rhode Island	4.17	699	34.6	28.2
South Carolina	3.19	791	44.8	36.4
South Dakota	4.32	257	43.2	35.8
Tennessee	3.73	1234	40.8	34.0
Texas	4.65	10281	45.5	37.8
Utah	3.92	1398	49.1	40.9
Vermont	3.82	220	35.5	30.5
Virginia	4.71	5642	36.0	28.4
Washington	4.71	3301	41.2	34.5
West Virginia	3.04	263	40.3	37.3
Wisconsin	3.93	1657	43.9	37.3
Wyoming	5.43	88	54.5	45.5
